# Prospective study of protein intake and mortality among US adults with chronic obstructive pulmonary disease

**DOI:** 10.3389/fnut.2024.1399038

**Published:** 2024-07-24

**Authors:** HuiLun Lu, Qi Zhang, Jiao Long

**Affiliations:** ^1^The Department of Respiratory Medicine, Shenzhen Longgang Second People’s Hospital, Shenzhen, Guangdong, China; ^2^Department of Anesthesiology, The Seventh Affiliated Hospital of Sun Yat-sen University, Shenzhen, Guangdong, China

**Keywords:** COPD, protein intake, mortality, multivariate cox, NHANES

## Abstract

**Background:**

Protein is crucial for the rehabilitation of patients with chronic obstructive pulmonary disease (COPD), and appropriate daily protein intake is essential for COPD patients. However, the specific role of protein intake in COPD and its impact on mortality remain uncertain. This study aims to ascertain the relationship between protein intake and mortality in COPD patients.

**Methods:**

This investigation included 522 adult COPD patients from the National Health and Nutrition Examination Survey (NHANES) between 2013 and 2018, with a focus on evaluating protein intake. Multivariate Cox proportional hazard models were constructed to analyze the correlation between protein intake and the prognosis of COPD patients. Additionally, the restricted cubic spline (RCS) was employed to investigate the potential non-linear association between protein intake and mortality.

**Results:**

A total of 522 patients with COPD were categorized into 4 groups based on the quartiles of protein intake: Q1 (< 25th percentile, 11.7–48.5 gm), Q2 (25–50th percentile, 48.5–67.7 gm), Q3 (50–75th percentile, 67.7–94.3 gm), and Q4 (≥ 75th percentile, 94.3–266.6 gm). Cox regression analysis revealed a significant trend in the p value of the Q3 group compared to the Q1 group when adjusting for other variables. The RCS-fitted Cox regression model indicated no non-linear relationship between protein intake levels and COPD mortality.

**Conclusion:**

There is no evidence of a non-linear relationship between protein intake and all-cause mortality in COPD patients. Further investigation is warranted to comprehend the intricate relationship between protein intake and COPD outcomes.

## 1 Introduction

Chronic obstructive pulmonary disease (COPD) represents a persistent and progressive inflammatory disorder affecting the airways, primarily characterized by irreversible airflow limitation and impaired ventilation (1, 2). As a leading contributor to global mortality and morbidity, COPD imposes a substantial economic burden on society (3). The influence of nutritional intake on the recovery of various diseases is well-established, and malnutrition exerts detrimental effects on disease outcomes.

Proteins, the principal constituents of cells, tissues, and organs, are vital for human health. Research has demonstrated that insufficient protein intake in patients with chronic respiratory diseases correlates with reduced physical activity and impaired lung function (4, 5). The effects of protein intake vary across different patient populations. In critically ill children admitted to pediatric intensive care units, adequate protein intake is associated with improved clinical outcomes and a decreased risk of mechanical ventilation (6). Previous studies have shown that in intensive care units, high protein intake is closely associated with the physical recovery of adult critically ill patients. Increasing protein intake not only significantly improves physical performance and muscle strength in these patients but also increases the recovery rate of independent walking (7, 8). These findings establish a crucial foundation for the management of clinical nutrition, stressing the importance of reasonable adjustment of protein intake during clinical diagnosis and treatment. In patients with prolonged mechanical ventilation, higher protein intake is associated with a shorter duration of ventilator use and successful weaning (9). However, in patients with diabetes, higher protein intake correlates with poorer glycemic control (10). The recommended protein intake for diabetic patients ranges from 10 to 20% of energy intake or 0.8–1.3 g/kg body weight, depending on age (11).

While some investigations have discovered that prioritizing energy and protein-rich foods may help improve the nutritional status and quality of life of COPD patients (12), others have reported insufficient protein intake may lead to an increased risk of exacerbating mild to moderate COPD (13). Nevertheless, certain research has identified a considerable correlation between pulmonary function and protein intake in COPD patients (14), with a notable association between protein intake extrapolated from food frequency questionnaires and forced vital capacity (FVC) and vital capacity (VC).

However, there is a paucity of literature on protein intake and all-cause mortality in COPD patients. This study sought to fill this gap by investigating the relationship between protein intake levels and COPD-related mortality using data from the National Health and Nutrition Examination Survey (NHANES). The analysis provides new insights into COPD treatment, laying the groundwork for further research.

## 2 Materials and methods

### 2.1 Study population

NHANES^1^ (15) is a cross-sectional survey based on the population, assessing the nutritional status and health of the US household population. In each 2-year study period, it randomly includes around 10,000 individuals. Data were acquired through interviews, covering socioeconomic, health-related, and demographic information, coupled with examinations, including medical assessments, laboratory tests, and physiological measurements conducted by trained medical personnel (16). NHANES has received approval from the National Center for Health Statistics Institutional Review Board, with all participants providing written informed consent (17). The survey aims to determine disease prevalence, identify risk factors, and provide essential data supporting the development of nutrition and health policies.

**FIGURE 1:**
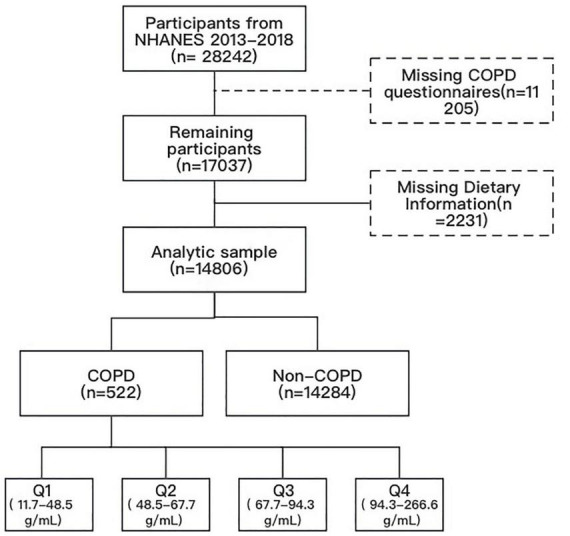
Flow chart.

This research amalgamated data from three NHANES cycles spanning 2013 to 2018. Our analysis encompassed all individuals aged 20 and over who participated in the COPD survey (responding affirmatively to the question “Has a doctor or other health professional ever told you that you had COPD?”) and had measured protein intake. Participants with missing covariate data were excluded A total of 522 COPD patients were included for further analysis (Figure 1).

### 2.2 Determination of protein intake

Dietary intake data were utilized to assess the varieties and quantities of beverages and foods (inclusive of all types of water) consumed within 24 hours preceding the interview (midnight to midnight). These data included energy and nutrient intakes, and other food components. The US Department of Health and Human Services (DHHS) and the National Center for Health Statistics (NCHS) oversees the survey design and all data collection, whilst USDA’s Food Surveys Research Group (FSRG) manages methods of data review and processing, databases maintenance, and dietary data collection. Protein intake was categorized into quartiles for statistical analysis: Q1 (< 25th percentile, 11.7–48.5 gm), Q2 (25–50th percentile, 48.5–67.7 gm), Q3 (50–75th percentile, 67.7–94.3 gm), and Q4 (≥ 75th percentile, 94.3–266.6 gm), with Q1 as the reference standard.

### 2.3 Mortality outcome

Death status and follow-up time information were obtained from the National Death Index in NHANES (as of May 2022). The cause of death was ascertained through the application of the International Classification of Diseases 10th Edition (ICD-10). The primary outcome of this study was all-cause mortality.

### 2.4 Covariates

Covariate factors encompassed age, gender (female, male), race (non-Hispanic whites and blacks, Mexican Americans, others), education level (less than 9th grade, 9–11th grade, high school graduate/GED or equivalent, some college or AA degree, college graduate or above), marital status (married or living with a partner, divorced or separated, widowed, and never married), family income to poverty ratio, smoking status (former, now, never), congestive heart failure (CHF, yes or no), alcohol (former, heavy, mild, moderate, never), diabetes (yes or no), hypertension (yes or no), BMI, coronary heart disease (CHD, yes or no), chronic kidney disease (CKD, yes or no), hyperlipidemia (yes or no), stroke (yes or no), atherosclerotic cardiovascular disease (yes, no), heart attack (yes or no), and study period 2013, 2014, 2015, 2016, 2017, 2018. Serum albumin (g/L), serum total protein (g/L) were considered as continuous variables.

### 2.5 Statistical analyses

Statistical analyses were performed using R version 4.2.1. COPD patients were stratified into four groups based on protein intake quartiles (Q1, Q2, Q3, Q4). Descriptive statistics were applied to analyze population characteristics, with appropriate sampling weights used. Continuous variables were compared by Kruskal-Wallis H test or analysis of variance, while chi-square tests were employed for categorical variables. A two-sided P-value below 0.05 was deemed statistically significant. Missing data were addressed through multiple imputations.

The relationship between protein intake and all-cause mortality was evaluated using Cox proportional hazard regression analysis. Hazard ratio (HR) and corresponding 95% confidence interval (CI) were calculated for three models: Model 1 (unadjusted), Model 2 (adjusted for age, gender, race, and education level, marital), and Model 3 (additionally adjusted for family poverty income ratio, smoking status, alcohol, diabetes, hypertension, BMI, CHD, CHF, CKD, hyperlipidemia, stroke, atherosclerotic cardiovascular disease, heart attack based on the factors adjusted in Model 2). Log-rank tests were used for statistical comparisons between groups according to protein intake.

Restricted cubic spline (RCS) models were established to explore the non-linear relationship between variations in protein intake and the risk of all-cause death in COPD patients, adjusting for the same factors as Model 3. If the non-linear relationship was identified, the Cox proportional hazard regression model was employed to identify an HR-related threshold point and validate the association between changes in protein intake both over and below the threshold and mortality.

## 3 Results

### 3.1 Baseline characteristics

A total of 522 participants with COPD were involved and categorized into four groups based on protein intake quartiles. Table 1 presents patient characteristics. Participants had an average age of 63 years, with 57% being females. The average protein intake among enrolled patients was 70gm. In comparison to the lowest quartile, participants exhibiting higher protein intake were inclined to be non-Hispanic White, male, and diabetic. No statistically significant variations were noted in biochemical indicators and other characteristics across the remaining groups (*P* > 0.05).

**TABLE 1:** Baseline characteristics according to the protein intake quartiles.

Characteristic	Overall	Q1 (11.7–48.5)	Q2 (48.5–67.7)	Q3 (67.7–94.3)	Q4 (94.3–266.6)	*p*-value
*N* (%)	522	131 (25.1)	130 (24.9)	130 (24.9)	131 (25.1)	
Age, years, mean (SD)	63 (54, 73)	61 (51, 73)	63 (56, 77)	63 (57, 72)	62 (48, 72)	0.2
Gender, *n* (%)						<0.001
Female	261 (57)	87 (67)	80 (74)	59 (53)	35 (35)	
Male	261 (43)	44 (33)	50 (26)	71 (47)	96 (65)	
Race, *n* (%)						0.016
Mexican American	34 (3.4)	6 (2.3)	11 (3.2)	5 (2.9)	12 (5.2)	
Non-Hispanic Black	85 (6.7)	21 (7.4)	25 (11)	18 (4.2)	21 (4.9)	
Non-Hispanic White	305 (78)	72 (77)	76 (76)	82 (77)	75 (81)	
Other Hispanic	44 (3.2)	20 (5.3)	9 (5.0)	4 (0.4)	11 (2.7)	
Other Race−Including Multi-Racial	54 (9.1)	12 (7.8)	9 (5.1)	21 (16)	12 (5.9)	
Education, *n* (%)						0.5
Less than 9th grade	52 (5.5)	19 (9.2)	15 (5.5)	7 (3.0)	11 (4.3)	
9-11th grade (Includes 12th grade with no diploma)	95 (14)	26 (16)	22 (13)	28 (18)	19 (7.6)	
High school graduate/GED or equivalent	147 (29)	38 (30)	37 (29)	30 (27)	42 (28)	
Some college or AA degree	164 (35)	38 (29)	39 (41)	46 (30)	41 (41)	
College graduate or above	64 (18)	10 (15)	17 (12)	19 (23)	18 (19)	
Marital, *n* (%)						0.4
Divorced	94 (13)	20 (12)	22 (13)	27 (16)	25 (11)	
Living with partner	30 (4.7)	7 (5.3)	10 (7.6)	6 (2.5)	7 (4.2)	
Married	236 (55)	55 (52)	52 (46)	57 (54)	72 (68)	
Never married	45 (6.2)	12 (5.4)	8 (3.9)	12 (8.7)	13 (6.0)	
Separated	26 (3.9)	8 (4.2)	7 (4.2)	6 (4.2)	5 (2.8)	
Widowed	91 (17)	29 (21)	31 (25)	22 (15)	9 (8.2)	
Family poverty income ratio, Mean (SD)	2.04 (1.05, 3.81)	1.93 (1.08, 3.75)	1.88 (1.02, 3.13)	2.10 (1.01, 3.67)	2.22 (1.21, 4.49)	0.4
Smoking status, *n* (%)						0.2
Former	83 (17)	17 (9.8)	25 (23)	23 (21)	18 (15)	
Never	255 (50)	59 (47)	63 (44)	66 (51)	67 (57)	
Now	184 (33)	55 (43)	42 (33)	41 (28)	46 (28)	
Alcohol, *n* (%)						0.3
Former	120 (21)	34 (25)	26 (14)	25 (14)	35 (30)	
Heavy	79 (17)	15 (14)	18 (16)	22 (17)	24 (22)	
Mild	193 (38)	40 (38)	51 (37)	52 (44)	50 (30)	
Moderate	81 (18)	24 (15)	25 (26)	18 (15)	14 (16)	
Never	49 (6.8)	18 (7.3)	10 (7.3)	13 (9.3)	8 (2.7)	
Diabetes, *n* (%)	240 (43)	63 (48)	45 (29)	69 (59)	63 (34)	<0.001
Hypertension, *n* (%)	367 (64)	93 (67)	87 (64)	90 (62)	97 (64)	>0.9
BMI, mean (SD)	30 (26, 34)	30 (26, 33)	30 (25, 34)	28 (25, 34)	30 (28, 38)	0.11
Protein, mean (SD)	70 (48, 93)	37 (29, 43)	58 (53, 62)	78 (73, 83)	118 (106, 151)	<0.001
Coronary heart disease, *n* (%)	82 (15)	29 (25)	15 (8.3)	25 (16)	13 (10)	0.042
Congestive heart failure, *n* (%)	87 (14)	28 (15)	19 (12)	19 (13)	21 (14)	>0.9
Chronic kidney disease, *n* (%)	186 (28)	47 (31)	45 (26)	43 (24)	51 (34)	0.5
Hyperlipidemia, *n* (%)	413 (83)	108 (81)	103 (85)	103 (82)	99 (82)	>0.9
Stroke, *n* (%)	65 (14)	15 (12)	14 (12)	17 (14)	19 (19)	0.7
Atherosclerotic cardiovascular disease, *n* (%)	172 (33)	51 (43)	40 (25)	40 (29)	41 (33)	0.2
Heart attack, *n* (%)	81 (14)	26 (19)	19 (12)	13 (6.7)	23 (18)	0.039
Serum albumin, Mean (SD)	41.0 (39.0, 43.0)	41.0 (39.0, 43.0)	41.0 (39.0, 42.6)	41.0 (38.0, 43.0)	42.0 (39.0, 43.0)	0.7
Serum total protein, Mean (SD)	69.0 (66.0, 72.0)	69.0 (66.5, 73.0)	68.4 (65.0, 72.0)	69.0 (66.0, 72.0)	68.0 (64.1, 71.0)	0.2

SD, standard deviation.

### 3.2 Multivariate cox proportional hazard regression analysis of dietary protein intake and its components in COPD

Out of the 522 patients, 100 deaths were recorded. The linear relationship between continuous protein intake and all-cause mortality in COPD patients was explored using three Cox regression models: Model 1, Model 2, and Model 3. No statistical significance was observed, with p values for Model 1, Model 2, and Model 3 at 0.8, 0.09, and 0.5, respectively (Table 2).

**TABLE 2:** HR with 95% CI for mortality based on the protein intake as a continuous variable.

	HR	95% CI	*p*-value
**All-cause mortality**			
Model 1	1.00	0.99, 1.01	0.8
Model 2	1.00	0.99, 1.01	0.9
Model 3	1.00	0.99, 1.00	0.5

Non-adjusted (Model 1). Adjusted for race, gender, age, education level, and marital (Model 2). Further adjusted for family poverty income ratio, smoking status, alcohol, diabetes, hypertension, BMI, coronary heart disease, chronic kidney disease, congestive heart failure, hyperlipidemia, stroke, atherosclerotic cardiovascular disease, heart attack based on the factors adjusted in Model 2 (Model 3); HR, hazard ratio; CI, confidence interval.

Subsequently, we adjusted for age, gender, race, education level, and marital status across the protein intake quartiles: Q1 (< 25th percentile, 11.7–48.5 gm), Q2 (25–50th percentile, 48.5–67.7 gm), Q3 (50–75th percentile, 67.7–94.3 gm), and Q4 (≥ 75th percentile, 94.3–266.6 gm). In Model 2, the all-cause mortality in the four groups was 1.00 (reference), 0.83 (95% CI: 0.41, 1.68; *P* = 0.6), 0.43 (95% CI: 0.21, 0.89; *P* = 0.22), 0.7 (95% CI: 0.32, 1.57; *P* = 0.4), respectively. In Model 3 further adjusted for family poverty income ratio, smoking status, alcohol, diabetes, hypertension, BMI, CHD, CHF, CKD, hyperlipidemia, stroke, atherosclerotic cardiovascular disease, and heart attack, the all-cause mortality in the four groups was 1.00 (reference), 0.93 (95% CI 0.49, 1.74; *P* = 0.8), 0.56 (95% CI 0.27, 1.16; *P* = 0.12), 0.69 (95% CI 0.32, 1.46; *P* = 0.3) (Table 3), separately.

**TABLE 3:** log (HR) (95% CI) for mortality according to the protein intake.

	Q1 (11.7–48.5)	Q2 (48.5–67.7)	Q3 (67.7–94.3)	Q4 (94.3–266.6)
**All-cause mortality**				
Number of patients	101	103	106	112
Model 1 HR(95% CI) *p*-value	1	1.08 (0.50, 2.32) 0.9	0.62 (0.28, 1.34) 0.2	0.82 (0.36, 1.85) 0.6
Model 2 HR(95% CI) *p*-value	1	0.83 (0.41, 1.68) 0.6	0.43 (0.21, 0.89) 0.022	0.70 (0.32, 1.57) 0.4
Model 3 HR(95% CI) *p*-value	1	0.93 (0.49, 1.74) 0.8	0.56 (0.27, 1.16) 0.12	0.69 (0.32, 1.46) 0.3

Non-adjusted (Model 1). Adjusted for age, gender, race, and education level, marital (Model 2). Further adjusted for family poverty income ratio, smoking status, alcohol, diabetes, hypertension, BMI, coronary heart disease, congestive heart failure, chronic kidney disease, hyperlipidemia, stroke, atherosclerotic cardiovascular disease, heart attack based on the factors adjusted in Model 2 (Model 3); HR, hazard ratio; CI, confidence interval.

Following adjustments for age, gender, race, education level, and marital status, no significant non-linear association was observed between protein intake and all-cause mortality, but a non-linear trend between the two was noted.

**FIGURE 2:**
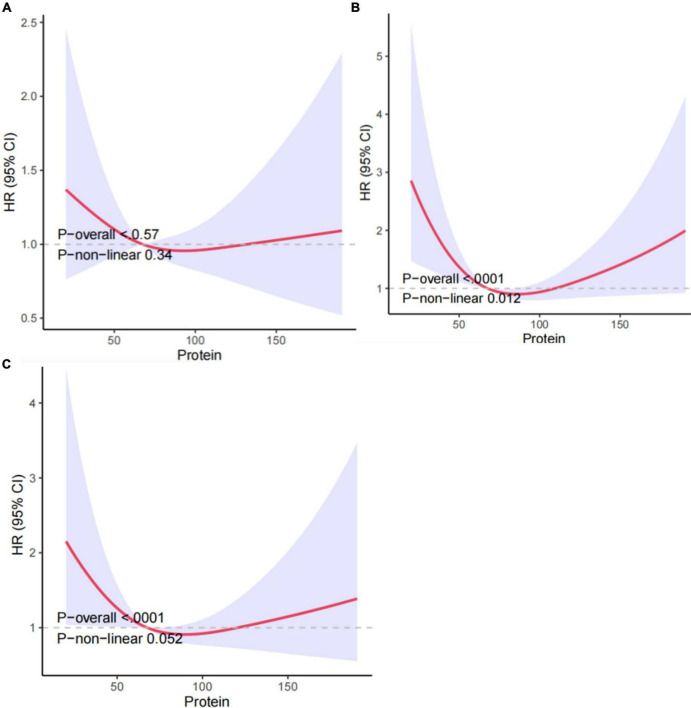
Association between protein intake and all-cause mortality in COPD patients: **(A)** non-adjusted Model 1, **(B)** Model 2 adjusted for gender, age, race, education level, and marital, **(C)** Model 3 further adjusted for age, gender, race, BMI, smoking, drinking, education level, hypertension, household income-poverty ratio, comorbidities and laboratory tests. HRs were calculated with the protein intake level of 62.8 as the reference. The solid line and the red region depict the estimated value and its corresponding 95% CI, respectively.

Therefore, we utilized RCS and smooth curve fitting to further examine the correlation. The adjusted smoothing map indicated no non-linear relationship between protein intake and all-cause mortality (Figure 2). In Figure 2B, the RCS curve analysis showed that after adjusting for gender, age, race, education level, and marital status, protein intake and all-cause mortality risk showed a U-shaped relationship. The lowest value was 90.3. It means that COPD patients have the lowest risk of all-cause mortality when the protein intake is 90.3gm. In Figure 2C, after further adjusting for BMI, smoking, drinking, household income-poverty ratio, comorbidities, and laboratory tests, the U-shaped relationship remained, but without statistical significance.

## 4 Discussion

This study represents the first prospective exploration of the relationship between protein intake and COPD mortality by analyzing COPD patients in the NHANES 2013–2018 cycle. The findings revealed no significant non-linear association between protein intake levels and mortality. Hence, further investigation is required to elucidate the intricate interplay between protein intake and mortality in COPD patients.

COPD stands as a significant global health concern that is anticipated to rank fifth in disease burden worldwide by 2020. It is estimated to ascend to the fourth most common cause of death by 2030 and the seventh in disability-adjusted life years (DALYs) loss globally (18). COPD has garnered growing attention from the medical community in recent years (19). Nutritional dietary protein intake has been a longstanding focal point of research in COPD, but the clinical understanding of its impact on the incidence, progression, and outcomes of COPD remains inadequately understood. Nowadays, there is a lack of studies examining the association between nutritional status and disease recovery or mortality in COPD patients. Nevertheless, research has identified a notable correlation between dietary protein intake and forced vital capacity (FVC) as well as vital capacity (VC) in patients with COPD (4). In general, patients with chronic respiratory diseases may undergo weight loss and accelerated muscle wasting during acute exacerbations, owing to malnutrition, reduced activity, hypoxia, systemic inflammation, and/or the combined effects of systemic corticosteroids (20). Thus, understanding the role of dietary protein intake in the management and prognosis of COPD becomes paramount.

Higher protein intake in COPD patients may be linked to the observed increased protein degradation rate. Evidence suggests elevated muscle protein degradation rates in COPD patients (21), characterized by heightened components of the ubiquitin 26S proteasome system (22) and an augmented role of autophagy (23). However, the potential repercussions of protein synthesis signal transduction remain unclear, specifically in response to catabolic triggers (24).

Protein intake in COPD patients has been reported to have varied effects. A study identifies insufficient protein intake in COPD patients referred for pulmonary rehabilitation (PR) (25), while others find no additional benefits in terms of muscle strength, physical function, and quality of life in non-sarcopenic COPD patients undergoing rehabilitation with the addition of protein supplementation (26). Another study discovers that energy and protein intake are below the calculated demand for all groups of COPD patients, revealing a notable correlation between protein intake and pulmonary function (27). Additionally, a study comparing the effects of a semi-solid snack and a liquid oral nutritional supplement (ONS) on energy, postprandial glucose, and protein intake reveals that postprandial glucose is elevated after consuming ONS compared to the snack after a meal (28). Overall, the role of protein intake in COPD patients is still under investigation, and further research is necessary to determine its optimal use in improving outcomes for these patients.

Nutritional interventions designed to stimulate protein synthesis and counteract increased protein degradation may help maintain muscle mass (29). Supplying adequate amino acids through nutritional interventions to support protein synthesis signaling could potentially induce a compensatory response to increased protein breakdown cues (30). Studies have indicated that the level of oxidative stress in the skeletal muscle of COPD patients is consistently elevated. In signaling pathways sensitive to oxidative stress and involved in the regulation of muscle mass, muscle biopsy analysis has demonstrated the activation of Forkhead box O (FoxO) (31), myogenin-activated protein kinase (MAPK) (32), and NF-κB (33). MAPK and NF-κB signaling is also induced by increased inflammation and inflammatory cell infiltration. A previous study has confirmed the expression of pro-inflammatory cytokines (34). Therefore, these catabolic pathways (or upstream triggers, such as oxidative stress and inflammation) may serve as potential targets for nutritional regulation (22).

The important implication of nutritional status for the prognosis of COPD patients has been widely recognized. Studies have shown that good nutritional status can significantly improve the quality of life, reduce acute exacerbations, and may prolong survival in COPD patients (35, 36). Muscle consumption and atrophy are closely related to the severity of COPD and affect the quality of life and survival rate of COPD patients. Muscle dysfunction is a prevalent issue among COPD patients, affecting about 20% to 35% of patients, especially those with severe symptoms or long-term bed rest. Muscle atrophy is related to the imbalance of muscle protein synthesis and decomposition, primarily attributed to factors such as inflammation, oxidative stress, hypoxia, and hypercapnia (37, 38).

Amino acid metabolism is also disrupted in the muscle tissue of COPD patients. One notable change is the decrease in the concentration of branched-chain amino acids (such as leucine), which are essential for providing energy to the muscle tissue. Leucine can not only provide energy for muscle tissue but also aids in the repair process after muscle injury. Therefore, disruptions in amino acid metabolism may exacerbate muscle damage and functional decline (39).

COPD primarily manifests as emphysema and chronic bronchitis, both of which have a significant impact on patient prognosis. Disease exacerbation is typically closely related to reduced quality of life, heightened hospitalization rate, and shortened life expectancy. Compared with mild or moderate COPD patients, patients with severe or extremely severe emphysema have a significantly elevated risk of all-cause mortality and cardiovascular mortality (40, 41).

Many factors affect protein intake, and previous research has underscored the importance of the proportion of protein in total energy intake. When considering total energy intake, an appropriate adjustment in protein intake could significantly impact health and longevity (42). Various research has indicated the significance of total energy intake on protein consumption. A thorough understanding of the fluctuations in energy requirements at different life stages is essential for devising appropriate strategies for proteins and other macronutrient intake to optimize health and function (43).

Although our RCS model and Cox regression analysis demonstrate no non-linear relationship between protein intake levels and mortality in COPD patients, this study has certain limitations. Initially, the absence of repeated protein intake measurements prevents the assessment of dynamic protein intake levels and their association with mortality. Second, the single-day recall method may not accurately reflect the regular food intake of the participants. Then, participants who self-reported ‘yes’ to the item “Ever told you had COPD?” may introduce some bias into the results, as we could not further validate the applicable diagnostic criteria. Additionally, despite adjusting for certain covariates in this study, other unadjusted covariates such as muscle wasting, sarcopenia, dynapenia, emphysema, and bronchitis might influence the mortality or protein intake levels. Consequently, a comprehensive prospective cohort study evaluating dynamic protein intake levels is crucial to corroborate the correlation between protein intake levels and COPD mortality.

## 5 Conclusion

In summary, this study, utilizing NHANES data, underscores the correlation between protein intake levels and all-cause mortality in COPD patients. No substantial non-linear correlation is observed between protein intake levels and all-cause mortality. Our results demonstrate that high protein intake does not have a protective effect in COPD patients. These results provide a theoretical basis for subsequent experimental verification and potential interventions for appropriate protein intake, offering new insights to improve the survival rate of this patient population.

## Data availability statement

Publicly available datasets were analyzed in this study. This data can be found here: https://www.cdc.gov/nchs/nhanes/.

## Author contributions

HL: Conceptualization, Data curation, Methodology, Software, Visualization, Writing−original draft. QZ: Conceptualization, Investigation, Methodology, Software, Writing−original draft, Writing−review and editing. JL: Conceptualization, Project administration, Supervision, Writing−review and editing.

## Footnotes

1
https://www.cdc.gov/nchs/nhanes/
